# Mapping Insertions, Deletions and SNPs on Venter's Chromosomes

**DOI:** 10.1371/journal.pone.0005972

**Published:** 2009-06-22

**Authors:** Maria Costantini, Giorgio Bernardi

**Affiliations:** Stazione Zoologica Anton Dohrn, Naples, Italy; Louisiana State University, United States of America

## Abstract

**Background:**

The very recent availability of fully sequenced individual human genomes is a major revolution in biology which is certainly going to provide new insights into genetic diseases and genomic rearrangements.

**Results:**

We mapped the insertions, deletions and SNPs (single nucleotide polymorphisms) that are present in Craig Venter's genome, more precisely on chromosomes 17 to 22, and compared them with the human reference genome hg17. Our results show that insertions and deletions are almost absent in L1 and generally scarce in L2 isochore families (GC-poor L1+L2 isochores represent slightly over half of the human genome), whereas they increase in GC-rich isochores, largely paralleling the densities of genes, retroviral integrations and *Alu* sequences. The distributions of insertions/deletions are in striking contrast with those of SNPs which exhibit almost the same density across all isochore families with, however, a trend for lower concentrations in gene-rich regions.

**Conclusions:**

Our study strongly suggests that the distribution of insertions/deletions is due to the structure of chromatin which is mostly open in gene-rich, GC-rich isochores, and largely closed in gene-poor, GC-poor isochores. The different distributions of insertions/deletions and SNPs are clearly related to the two different responsible mechanisms, namely recombination and point mutations.

## Introduction

The very recent availability of fully sequenced individual human genomes [1]–[5] is a major revolution in biology which is certainly going to provide new insights into genetic diseases and genomic rearrangements in the near future. In the present work, we looked at the insertions, deletions and SNPs that are present in Craig Venter's genome [1], more precisely on chromosomes 17 to 22 (334 megabases, about 10% of the human genome), and compared them with the human reference genome hg17 from UCSC website.

The three main reasons for carrying out this investigation were the following: (i) to localize insertions, deletions and SNPs on chromosomes 17 to 22, in connection with the compartmentalization of the human genome into isochores [6], [7]; this was done at two levels, namely localization in isochore families (L1, L2, H1, H2, H3, in order of increasing GC and gene density) and mapping within the isochores; (ii) to correlate insertions, deletions and SNPs with the densities of genes, interspersed repeats and retroviral insertions, since these densities are correlated, in turn, with isochore GC levels [8]–[12], [6], and since they may provide indications for the preference of insertions/deletions for different isochore families; (iii) to prepare the ground for exploring the expression of genes located in the neighborhood of deletions and insertions; indeed it has been postulated [7] that compositional changes due to the accumulation of AT-biased point mutations or to deletions/insertions may be associated with alterations of chromatin structure that, in turn, may affect gene expression.

It should be pointed out that the present work only concerns (i) insertions and deletions among structural variations (not including copy-number variations such as segmental duplications; see ref. [13] for a review, and ref. [14]); and (ii) SNPs as detected by pairwise alignment of sequences. It should also be stressed that the Venter genome used in our comparison, represents a composite haploid version of the genome where the highest scoring alleles contained are represented in the consensus sequence. The human reference genome hg17 (practically identical to the latest hg18 version for the chromosomes under consideration) is a composite genome resulting from several individuals. Insertions and deletions, as well as SNPs, reported in this article are, therefore, the result of the comparison of one genome, the Venter genome, with several individual genomes. In other words, each insertion and deletion in Venter is derived from a comparison with another individual, but not necessarily the same individual. Obviously, this also applies to SNPs. We thought that our approach was acceptable in view of the fact that our primary aim was to look for the localization of insertions/deletions and SNPs on isochores.

Focusing on chromosomes 17–22 is justified by considering that these chromosomes are representative, in terms of isochores, of the whole human genome. A detailed comparison of the full Venter genome with the human reference genome was not warranted at the time of our investigations, because the human reference genome, as already mentioned, is a composite genome. Obviously, a comparison of full individual genomes will be of interest as soon as this will be possible.

## Results

**Figure 1:**
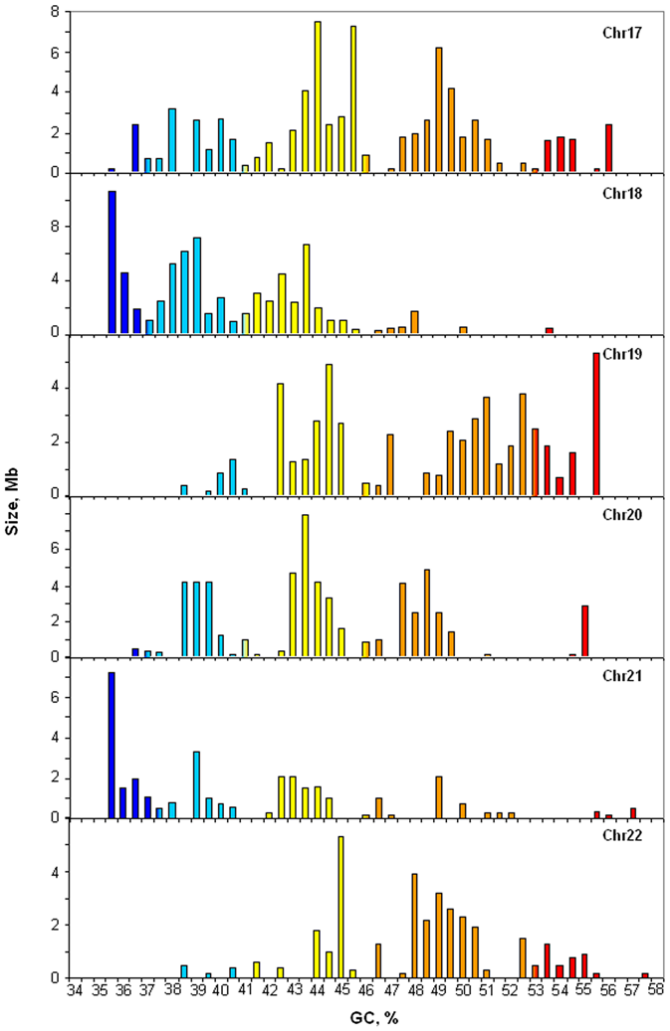
Distribution of isochores on chromosomes 17 to 22 from the human reference genome. The histograms show the distribution (by weight) of isochores as pooled in bins of 0.5% GC for chromosomes 17 to 22 from hg17. Colors represent the five isochore families. The color code spans the spectrum of GC level in five steps, indicated by broken horizontal lines: ultramarine blue (L1), light blue (L2), yellow (H1), orange (H2) and red (H3). Note the different scales on the ordinate axis.

The choice of chromosomes 17 to 22 was due to the fact that while these chromosomes exhibit wide differences in their isochore patterns, they cumulatively show an overall similarity with the isochore patterns of the whole human genome [15]. Indeed, as shown in Figure 1, chromosomes 17 and 20 are characterized by a predominance of H1 and H2 isochores, whereas L1 isochores are poorly represented. In contrast, chromosomes 18 and 21 are characterized by abundant L1 isochores (as well as L2 isochores in the case of chromosome 18, which lacks H3 isochores altogether). Chromosomes 19 and 22 completely lack isochore family L1, are very scarce in L2 isochores, and show a great abundance of H1 and, especially, of H2 isochores. It should be noted that while Figure 1 reports the isochore patterns of chromosomes from release hg17, the isochore profiles of hg17 and hg18, the most recent release, are identical as far as chromosomes 17 to 22 are concerned, the only exceptions being three small gaps in hg17 of chromosome 22 which were filled in the hg18 version (see Figure S1).

**Figure 2:**
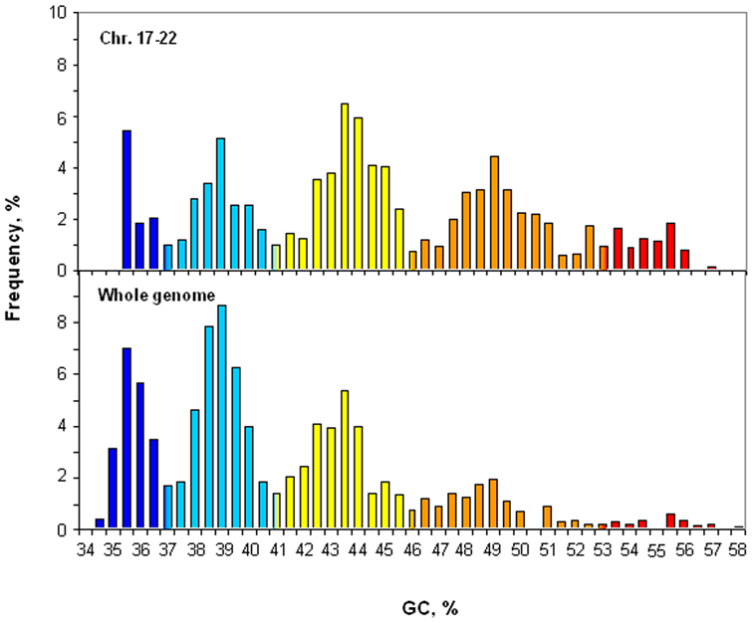
Comparison of the cumulative isochore distribution on chromosomes 17 to 22 and on the whole human genome. The isochore distribution of the whole human genome is from ref. 15. In order to compare the two histograms, isochore frequencies were calculated as percentages of the total. The color code spans the spectrum of GC level in five steps, indicated by broken horizontal lines: ultramarine blue (L1), light blue (L2), yellow (H1), orange (H2) and red (H3).

Figure 2 compares the cumulative isochore pattern of chromosomes 17 to 22 with that of the whole human genome. The former one is characterized by an under-representation of GC-poor isochore families L1 and L2 and by an over-representation of GC-rich isochore families H1, H2 and H3. Chromosomes 17 to 22 still provide, however, a fair representation of the isochore pattern of the whole human genome, which is satisfactory for the purpose of this investigation. In addition, these differences are take care of the fact that our data on insertions/deletions are presented as densities.

**Figure 3:**
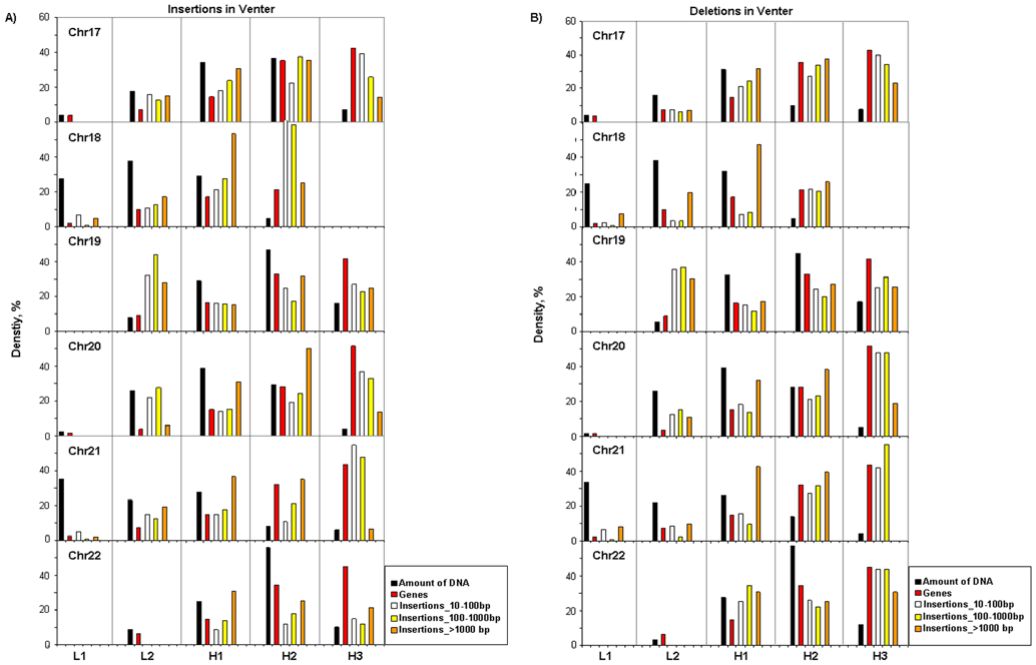
Insertions/deletions in Venter's chromosomes. For each chromosome the amounts of DNA (in percentage of the total; black bars) and the densities of genes (red bars), insertions (A) and deletions (B) (in the three size classes 10–100 bp, 100–1000 bp, >1000 bp; white, yellow and orange bars) are reported for the five isochore families of Venter's chromosomes. The slightly different amounts of DNA in isochore families between (A) and (B) are related to the fact that deletions in Venter's chromosomes are seen as insertions in the reference chromosomes, and the latter are slightly different from Venter's chromosomes because of insertions and deletions. In some cases in which DNA amounts are very low (such as in L1 of chromosomes 17 and 20, and L2 of chromosomes 22) the insertion/deletion densities were not reported (see, however, Supplementary Table S1).

**Figure 4:**
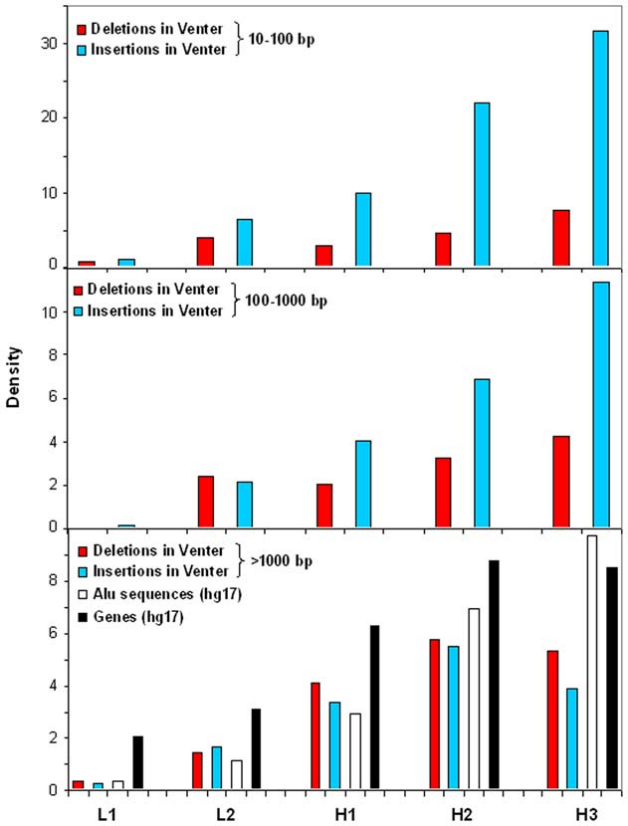
Density of insertions and deletions in isochore families from chromosomes 17 to 22. The densities of insertions/deletions in chromosomes 17 to 22 are reported for the five isochore families. For the sake of comparison, Alu and gene densities (divided by 100 and by 4, respectively) in hg17 are also reported.

The locations of insertions and deletions, respectively, in the isochore families of Venter's chromosomes 17 to 22 are summarized in Figure 3 A,B. The correlation between the number of indels and proportion of sequence in isochors were determined using the Pearson correlation coefficient: very significant values (P<0.0001) were found. Densities of insertions and deletions in the three size ranges explored were extremely low in L1 isochores. While this is hardly surprising for chromosomes 19 and 22, which comprise few or no L1 isochores, this is also true for chromosomes 18 and 21, which are rich in L1 isochores. The density of insertions/deletions increased with increasing GC of isochore families, essentially paralleling the densities of genes and *Alu* sequences, except for the lower values of the longest (>1000 bp, base pairs) insertions/deletions in H3 isochores. In addition, in the latter case deletions and insertions showed a parallel behaviour, whereas insertions in Venter's chromosomes were more abundant than deletions in H1 to H3 families for the 10–100 and 100–1000 bp classes. The points made above expectedly appear more clearly on the cumulative plots of Figure 4.

It should be pointed out that (i) if the Venter genome contains two contiguous Alu elements (∼600 bp), while the human reference genome contains one Alu element (∼300 bp) at the orthologous locus, this locus will be assessed as a Venter genome insertion; and (ii) Alu-Alu recombination-mediated deletions (ARMDs) have been shown to occur frequently throughout primate evolution [16], [17]. Therefore, if this locus was created by an ARMD event in the human reference genome, one should discard this locus in the Venter insertion category. While this is correct in our case, ARMD's could only represent 50 human specific deletions (10% of the 492 found by Sen et al., 2006, for the whole genome since Venter's chromosomes 17 to 22 that represent 10% of the human genome). This is, however, a negligible number compared to the 3468 insertions in Venter found by us and would therefore not change our conclusions.

The results in terms of numbers of insertions/deletions located in different isochore families are reported in Table S1, which also presents the corresponding amounts of DNA. The data show (i) that the predominant weight contribution (>90%) expectedly is that of the largest insertions/deletions; (ii) that the total amounts of both insertions and deletions represent 0.6–2.7% of chromosome sizes, except for the much larger levels in the case of chromosome 19 (3.9% and 12.1%, respectively, for insertions and deletions in Venter); and (iii) that, in general, the patterns of deletions and insertions tend to parallel each other, with the exception of the very abundant deletions in Venter's chromosome 19.

**Figure 5:**
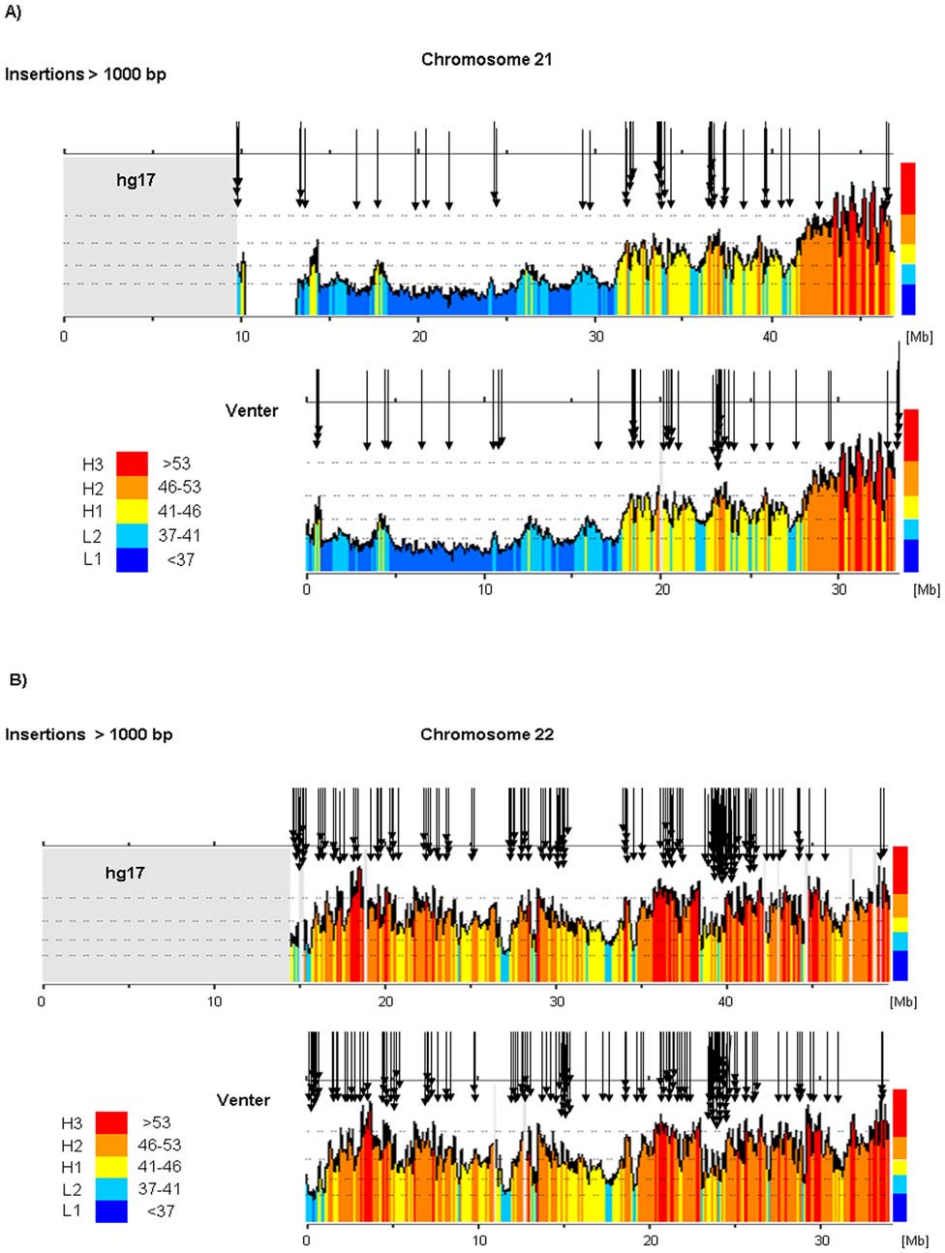
The largest insertions/deletions in chromosomes 21 and 22. Localizations of insertions and deletions larger than 1000 bp in chromosomes (A) 21 and (B) 22, are represented by the black arrows. The large gray blocks present in the hg17 diagrams are due to calculating the GC level using the program draw_chromosome_gc.pl (http://genomat.img.cas.cz; [49], [50]) that inserts grey lines or blocks where there are gaps. The telomere regions were presented as gaps in hg17 but eliminated in the Venter genome.

The localizations of insertions/deletions larger than 1000 bp in chromosomes 21 and 22 are showed in Figure 5. Two features are outstanding (i) the practical absence of insertions and deletions in sub-telomeric regions (e.g. positions 40 to 47 megabases on chromosome 21 of hg17), in spite of the fact that these regions are very GC-rich; and (ii) the highest concentrations of insertions/deletions in regions about position 37 megabase in chromosome 21 of hg17, and about position 39 megabase in chromosome 22 of hg17. These regions do not show any noticeable difference, in the present state of knowledge, when compared with compositionally similar regions located elsewhere on the chromosomes. The localizations of insertions/deletions of 10–100 bp and 100–1000 bp on chromosomes 21 and 22 are reported in Figures S2 and S3.

The parallelism between the densities of insertions and *Alu* sequences prompted a search for *Alu* sequences in the insertions of the reference human chromosomes that correspond to deletions in Venter's chromosomes. The results, presented in Table 1, indicate that all or most *Alu* sequences were present at the ends of 10–100 and 100–1000 bp insertions, respectively, whereas only about 30% of the >1000 bp insertions had *Alu* sequences at their ends, the majority of *Alu*s being located in internal positions.

**Table 1:** The number and locations of Alu sequences are reported for three classes of insertions (10–100 bp, 100–1000 bp and >1000 bp) in the human reference genome(a).

	Number	Locations of Alu sequences	
		Ends	Internal
**10–100 bp**	299	298	1
**100–1000 bp**	1734	1629	105
**>1000 bp**	888	246	642

(a) Locations of Alu sequences in Venter' s chromosomes are not reported because the coordinates for Alu sequences are not available.

**Figure 6:**
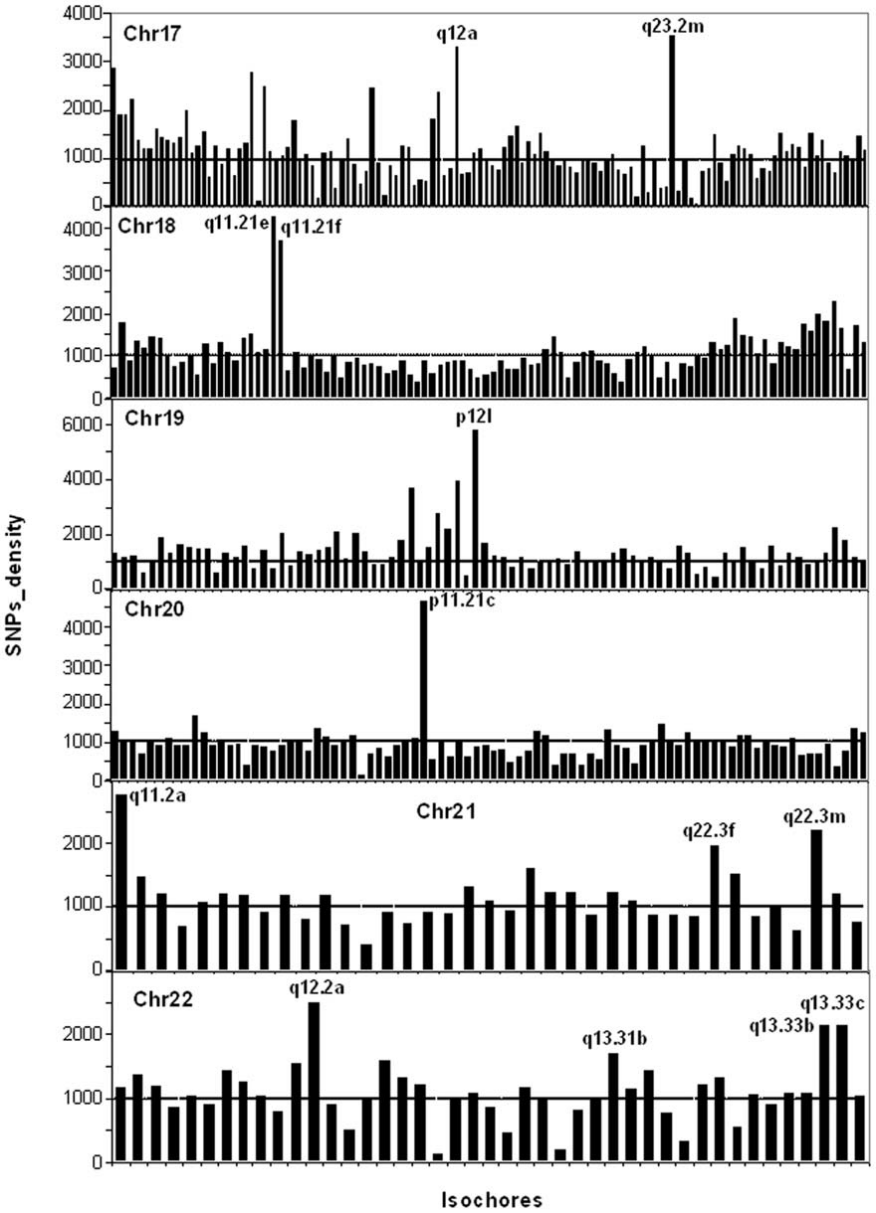
SNPs densities in isochores of chromosomes 17–22. Each bar corresponds to an isochore. The names of isochores with high densities of SNPs are reported. For the coordinates and the nomenclature of the other isochores see Supplementary Table S1 of ref. 15. The horizontal broken line at a density of 1000 corresponds to the average density of SNPs per megabase (see also Table S2). Supplementary Figure S6 presents the numbers of SNPs on the same chromosomes.

**Figure 7:**
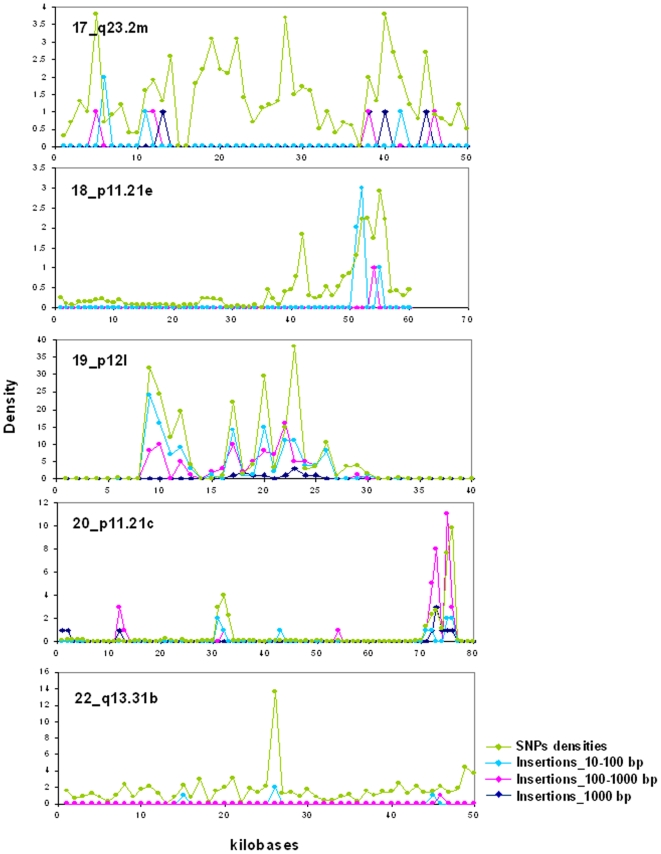
Insertions and SNPs in individual isochores of chromosomes. Numbers of insertions (in the three size classes 10–100 bp, 100–1000 bp, >1000 bp indicated by light blue, pink and dark blue, respectively) and densities of SNPs are reported for some of the isochores that show high densities of SNPs (see also legend of Figure 6).

In sharp contrast with insertions/deletions, the densities of SNPs were largely uniform over all isochore families (Figure 6; see also Table S2; Figure S4 presents the numbers of SNPs on chromosomes). Even if the vast majority of isochores showed relatively constant concentrations of SNPs, which did not vary with the different GC levels of isochores, a small number of them showed very high or very low concentrations (see Figure 6). When these isochores were analyzed individually (see Table S3), the high SNPs concentrations were found to be either distributed over most of the isochore length (as is the case for isochores having the average SNPs concentration) or present in limited regions (see Figure 7, in which five isochores are reported; for the other isochores see Figure S5). Insertions, being much less numerous than SNPs, were expectedly less widespread in their distribution and tended to coincide with SNPs spikes.

**Figure 8:**
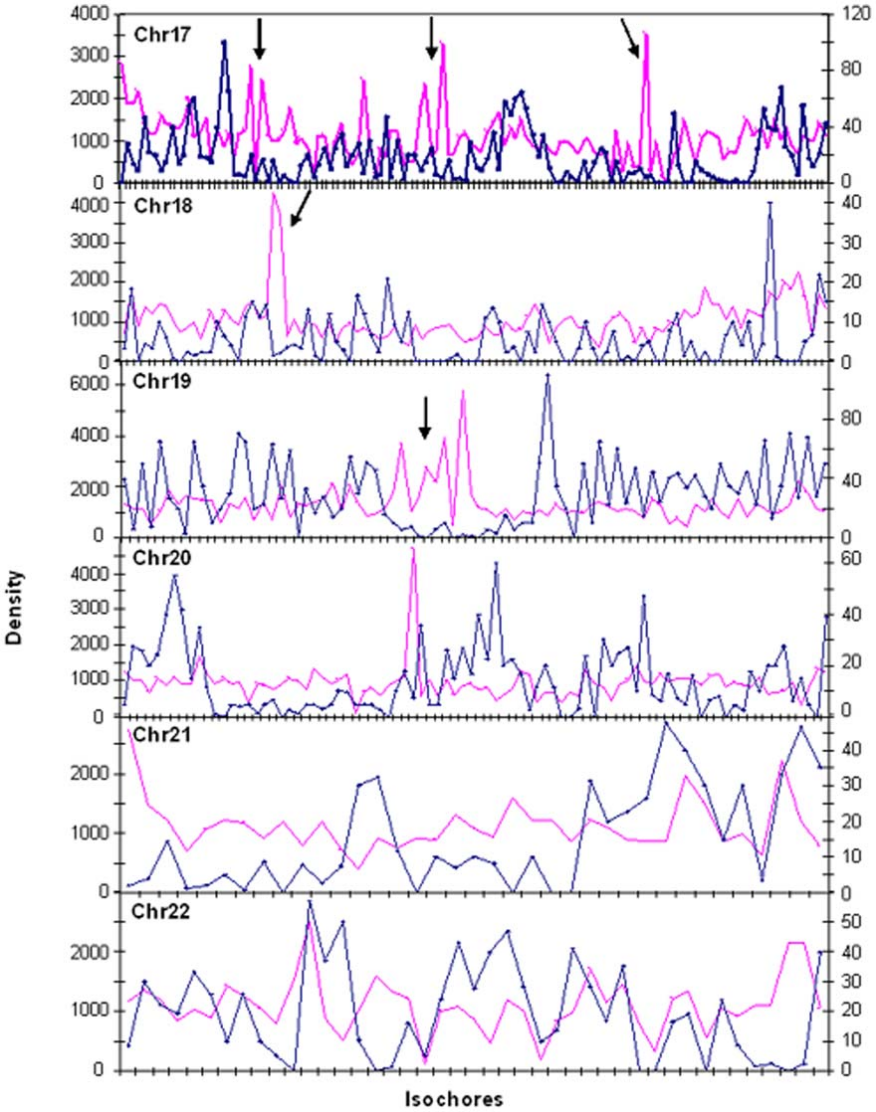
SNPs and gene densitites. Each point corresponds to an isochore. SNPs density (pink line) is compared with gene density (blu line) for chromosomes 17–22. Arrows show some of the opposite trends of densities. For the coordinates and the nomenclature of isochores on x-axes see Supplementary Table S1 of ref. 15 (see also legend to Figure 6).

Finally, a trend to avoid gene dense regions was evident when comparing gene density and SNPs density (Figure 8). P values <0.0001 were found for the correlation between gene density and SNPs density.

## Discussion

The most relevant result of the present investigation concerns the large preference for both insertions and deletions to take place in GC-rich isochores, especially in the H2 and H3 families, which only represent together 15% of the human genome.

The increase in insertions and deletions in the H1-H3 isochore families, parallels the increase in the concentration of both *Alu* sequences and genes (see Introduction), as well as in the degree of “openness” of chromatin [18]–[20] and in the frequency of recombination [21]–[25]. The question should therefore be asked which one(s) of these factors is (are) the most biologically significant as an explanation for the distribution of insertions/deletions.

The correlation between the densities of insertions/deletions and *Alu* sequences is indicated in the most evident way by the terminal distribution of *Alu* sequences in insertions in the reference human genome (see Table 1). While such terminal distribution is perfect for the 10–100 bp insertions and still predominant for the 100–1000 bp class, this is not, however, the case for the largest insertions, where *Alu* sequences are in terminal positions of only about 30% insertions. The distribution of insertions/deletions in GC-rich isochores is, however, not simply due to their richness in repeated sequences such as *Alu* sequences. Indeed, if this were the case, one would expect to have high levels of insertions/deletions also in GC-poor isochores, which are very rich in the other major family of interspersed repeats, the LINE-1 (long interspersed element-1) family, whereas this is not the case.

An overall positive correlation also exists between insertions/deletions and gene density but the longest insertions/deletions decrease in the most gene-dense isochores of the H3 family, as if this process were not allowed because of its deleterious impact on genes; and (ii) the insertions/deletions of the other size classes are scarce in telomeric regions, which are very gene-rich, as compared with similarly GC-rich, but less gene-rich isochores located elsewhere on chromosomes. At this point, one should conclude that the correlation between insertions/deletions and gene density is only a consequence of the correlation between gene density and GC level [6].

Having ruled out gene concentration as a factor favoring insertions/deletions (in fact, the opposite being true), and considering that *Alu* sequences are simply used in the recombination process (LINE-1 not favoring insertions/deletions in GC-poor isochores), the possibility remains that the real reason for the distribution of insertions/deletions reported here is the different chromatin structure of GC-poor vs GC-rich isochores [18]–[20]. This possibility is strongly supported by previous work on retroviral integration.

Indeed, Bovine Leukemia Virus (BLV; [26]), Human Hepatitis B (HBV a DNA virus with some retroviral features; [27]), Rous Sarcoma Virus (RSV; [28]), Human T-cell Leukemia Virus [29]), Murine Leukemia Virus (MuLV; [30]) were all shown to integrate in GC-rich isochores (see [6] for a review). One might, however, argue that, since all the retroviral sequences mentioned so far are GC-rich [31], integration into GC-rich isochores could depend upon the requirement for a compositional match between the retroviral sequence and the isochores of the host genome without being related to chromatin “openness”. Integration into GC-rich isochores was also found, however, for exogenous Mouse Mammary Tumor Virus (MMTV; [32]) and Human Immunodeficiency Virus (HIV-I; [6], [33]–[36]) which are GC-poor. This obviously favors the idea of an integration into open chromatin structures. Moreover, using different approaches, several authors [37]–[42] found high frequencies of RSV, Avian Leukosis Virus (ALV), and MuLV near DNase-hypersensitive sites, transcriptionally active regions and CpG islands. These results are in agreement with our conclusion since GC-rich isochores correspond to open chromatin regions [23] and since DNase-hypersensitive sites are concentrated in GC-rich isochores [24], [25] which are rich in genes and in CpG islands and are transcriptionally active. In conclusion, the results available indicate that the initial integration of retroviral sequences takes place in open chromatin regions (such as those corresponding to GC-rich isochores), whereas stability of integration and transcription requires a matching composition of retroviral and host sequences [6], [18]. Another result in favor of the open chromatin interpretation is that “new” *Alu* sequences integrate essentially at random in the genome, but this happens in the paternal germ line [43]–[45], where open chromatin is much more widespread over chromosomes.

At this point one should recall that the pattern of insertions/deletions follows the general pattern of chromosomal rearrangements [18] and recombination [20]–[22]. This might be an alternative possible explanation for the pattern of insertions/deletions. It seems, however, much more plausible that the pattern of recombination itself is dependent upon the distribution of open chromatin regions over the genome. Indeed, DNA duplications also occur more frequently in GC-rich compared to GC-poor isochores [44] and chromosomal fission takes place frequently within regions elevated in GC [46]. As already mentioned, in several cases the localizations of insertions/deletions in chromosomes indicate some specific preferences, such as those shown in Figure 5 and Table S1, which correspond to hot spots of recombination.

These observations are important because structural genome variations, such as insertions/deletions, may be involved in genetic diseases. We have already suggested that this may occur not so much through a direct impact on genes, but rather through local changes in chromatin structure that affect gene expression at a distance [7]. This explanation is supported by the fact that non-coding sequences are so overwhelmingly abundant compared to coding sequences in the human genome (98–99% vs 1–2%; [6]).

In sharp contrast with insertions/deletions, SNPs are rather uniformly distributed over all isochore families. The distribution of SNP is understandable because the main cause of SNPs are point mutations due to errors during DNA replication, which are apparently not very sensitive to the compositional context. Still, even if this applies to the vast majority of isochores, a small number of them showed very high or very low concentrations. Needless to say, the latter isochores deserve further investigation, also because of the coincidence of recombination hot spots and high SNP densities as shown by Figure 7 and Figure S6.

## Methods

Venter's chromosomes were downloaded from GenBank (http://www.ncbi.nlm.nih.gov/GenBank; accession number ABBA01000000; [1] and were aligned with the human reference genome hg17 [47], [48] on the UCSC website http://genome.ucsc.edu). This release, used for the mapping of isochores by Costantini et al. [15] was compared with the most recent release hg18, and found to be identical as far as chromosomes 17 to 21 are concerned, whereas chromosome 22 showed three small gaps, which were filled in the hg18 version. A script implemented by us was used to align the sequences and to extract the insertions/deletions in Venter's chromosomes, considering three size classes (10–100, 100–1000, >1000 bp), as well as the single nucleotide polymorphisms (SNPs). Insertions/deletions of single nucleotides in Venter's genome were also estimated and represented less than 5% of SNPs. *Alu* sequences coordinates for human genome reference were downloaded from UCSC website.

The correlations between the number of indels and proportion of sequence in isochores and between gene density and SNPs density were determined using the Pearson correlation coefficient by the statistical program Prism 4 (GraphPad Software San Diego, CA, USA). A value of P<0.05 was considered to be statistically significant.

## Supporting Information

Figure S1 (0.02 MB PDF)

Figure S2 (0.05 MB PDF)

Figure S3 (0.07 MB PDF)

Figure S4 (0.04 MB PDF)

Figure S5 (0.08 MB PDF)

Table S1 (0.03 MB XLS)

Table S2 (0.02 MB XLS)

Table S3 (0.02 MB XLS)
