# Supplementary figures and images for: Mapping Insertions, Deletions and SNPs on Venter's Chromosomes
Source: PLoS One. 2009 Jun 22;4(6):e5972. doi: 10.1371/journal.pone.0005972 (PMC2696090; doi:10.1371/journal.pone.0005972)

Supplementary Fig. S1

Chromosome 22

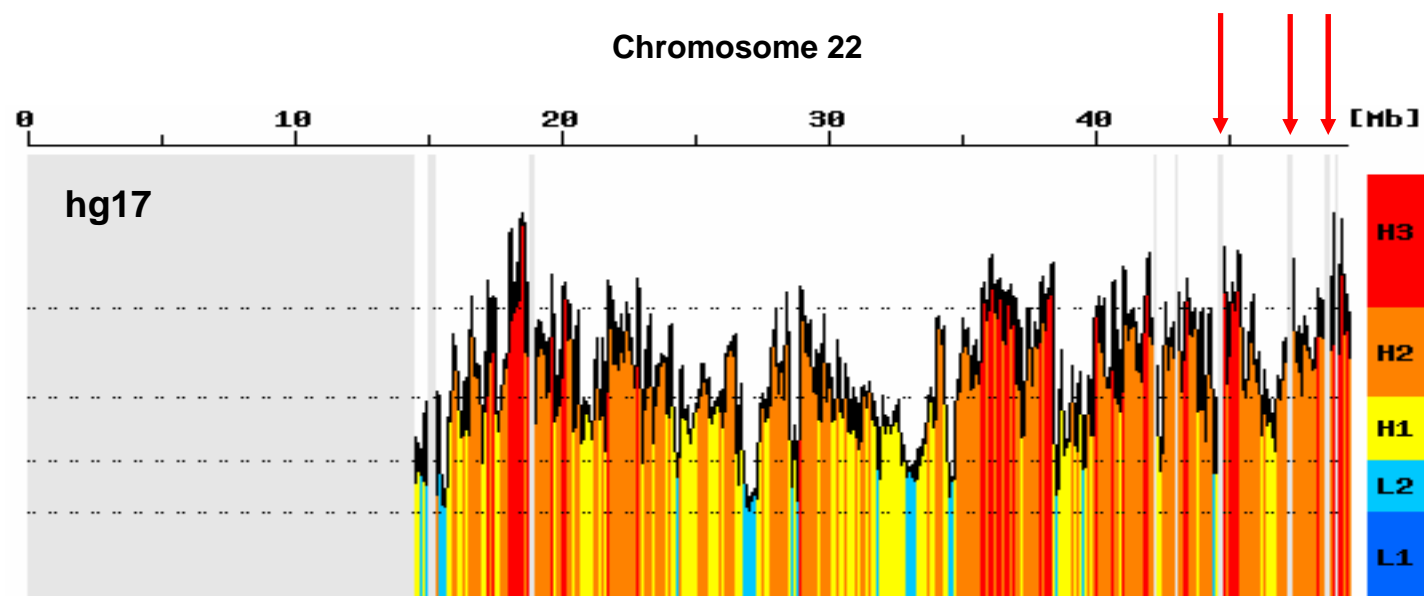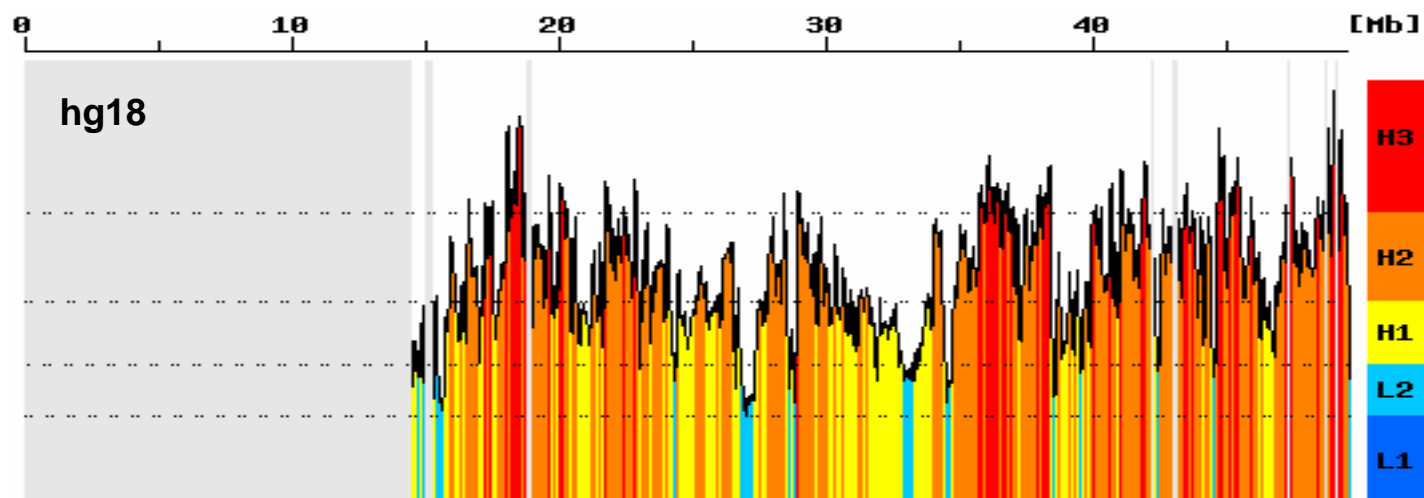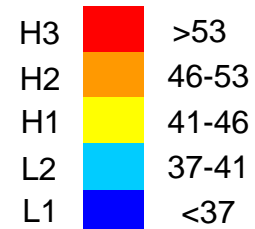

Supplement: Figure S1 — (0.02 MB PDF) [file pone.0005972.s001.pdf]

Suppl. Fig. S2A

Chromosome 21

Insertions 10 -100 bp

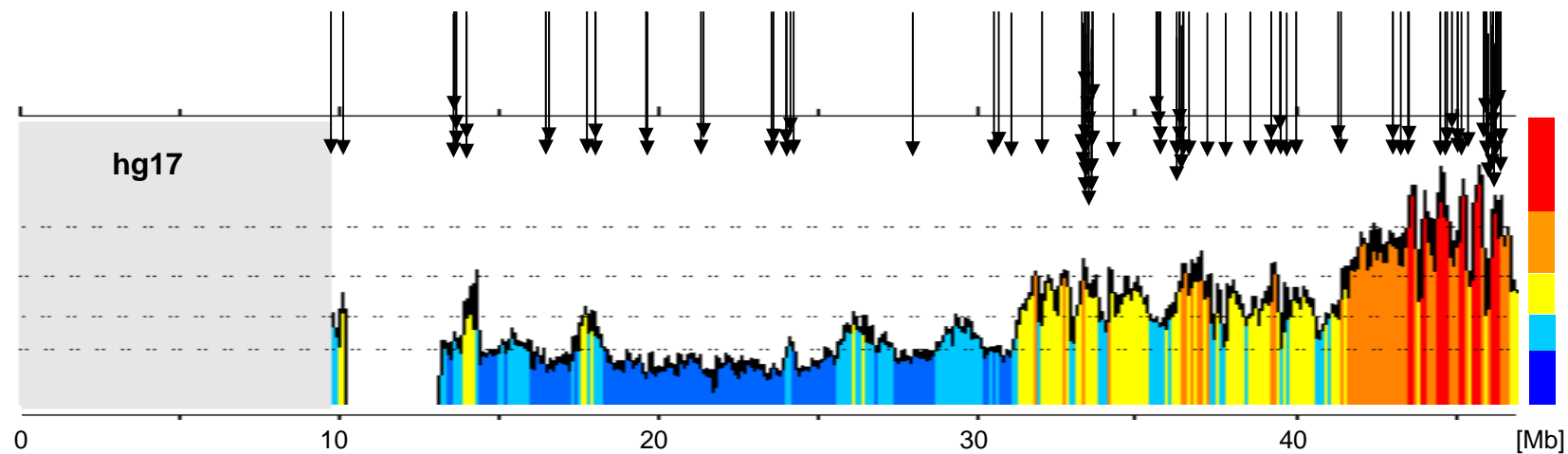

Venter

H3 >53  
H2 46-53  
H1 41-46  
L2 37-41  
L1 <37

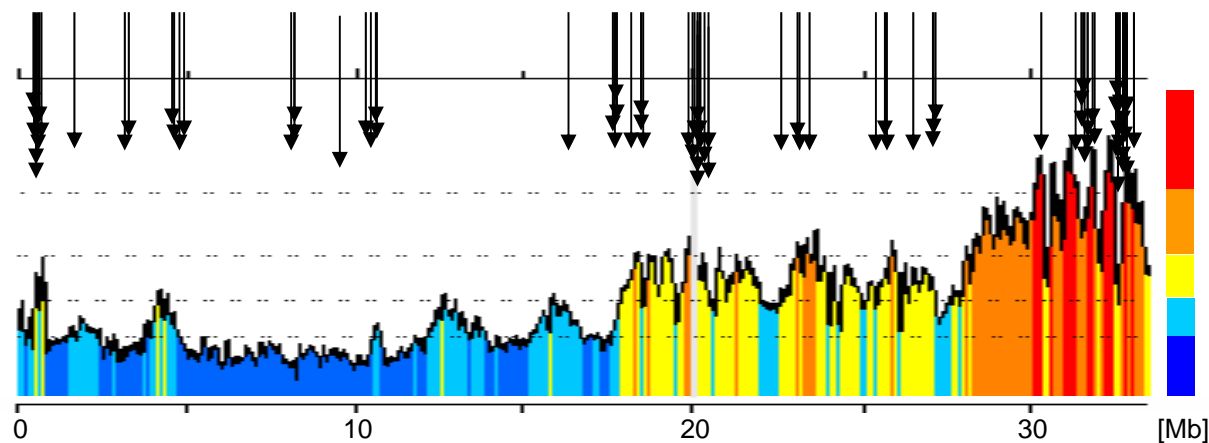

Suppl. Fig. S2B

Chromosome 21

Insertions 100-1000 bp

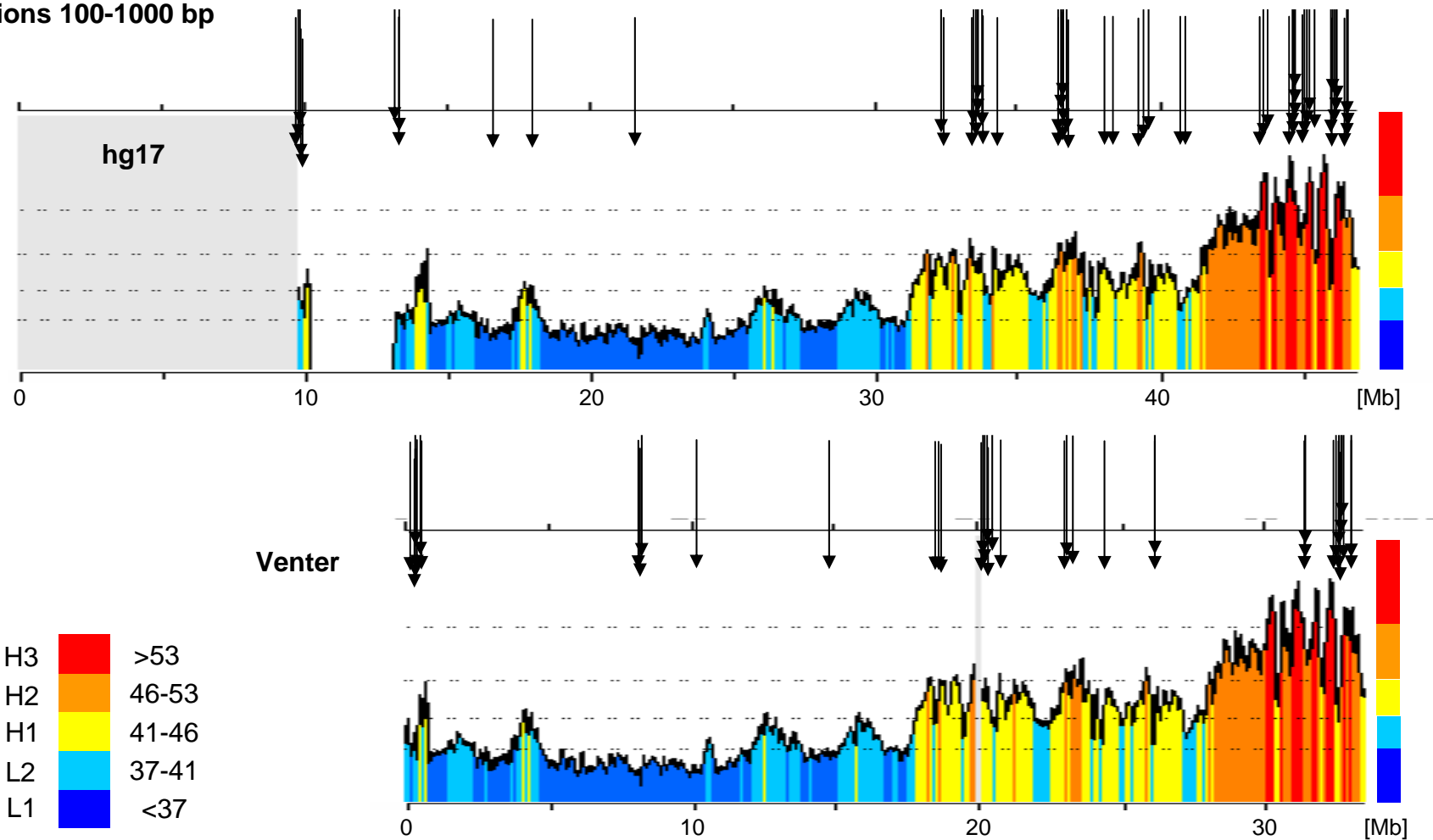

Supplement: Figure S2 — (0.05 MB PDF) [file pone.0005972.s002.pdf]

Suppl. Fig. S3A

Chromosome 22

Insertions 10 -100 bp

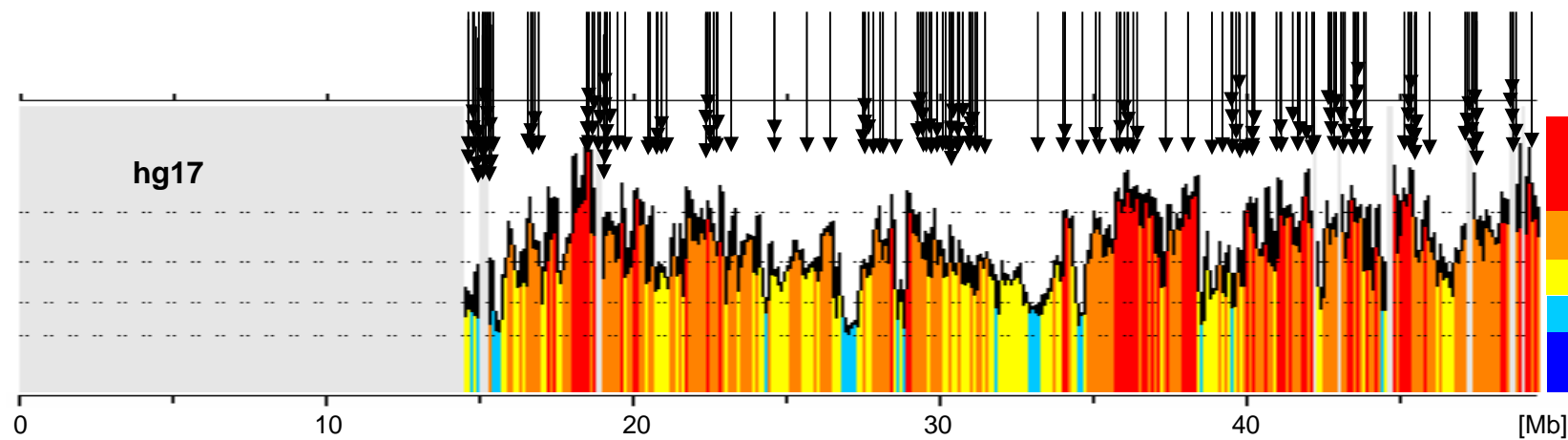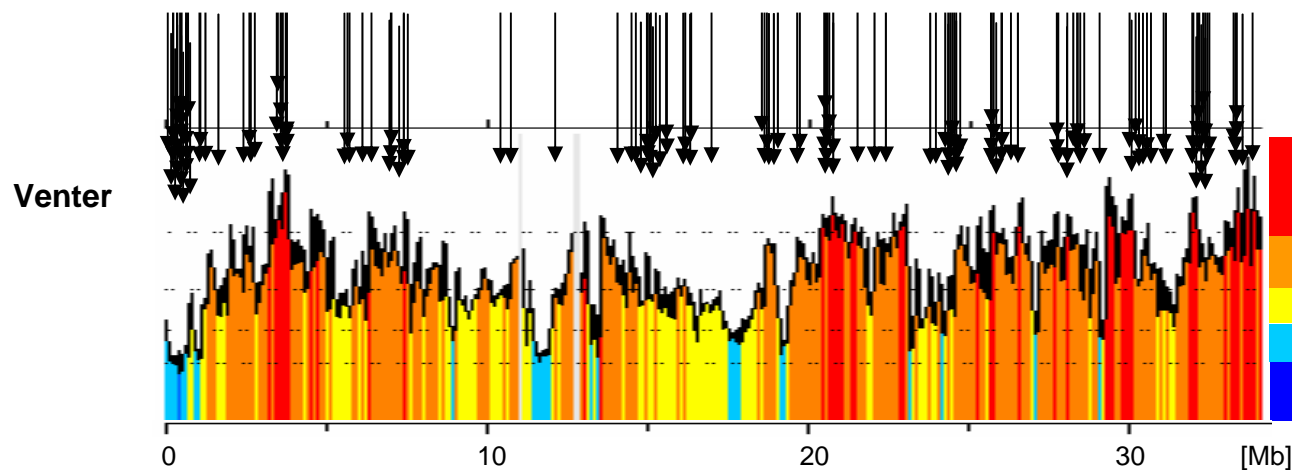

Suppl. Fig. S3B

Chromosome 22

Insertions 100 -1000 bp

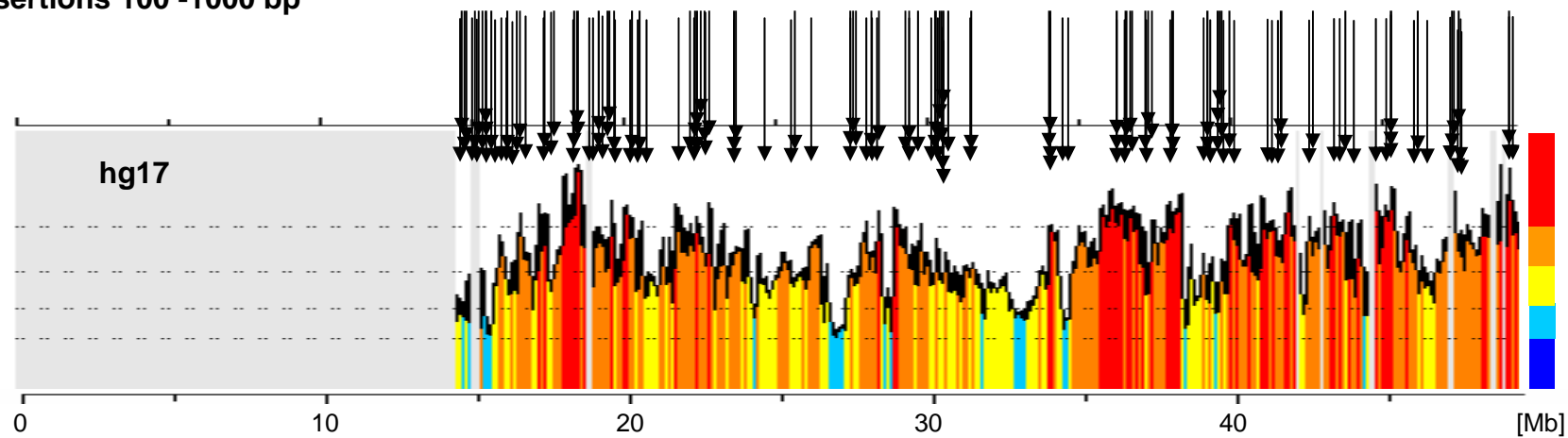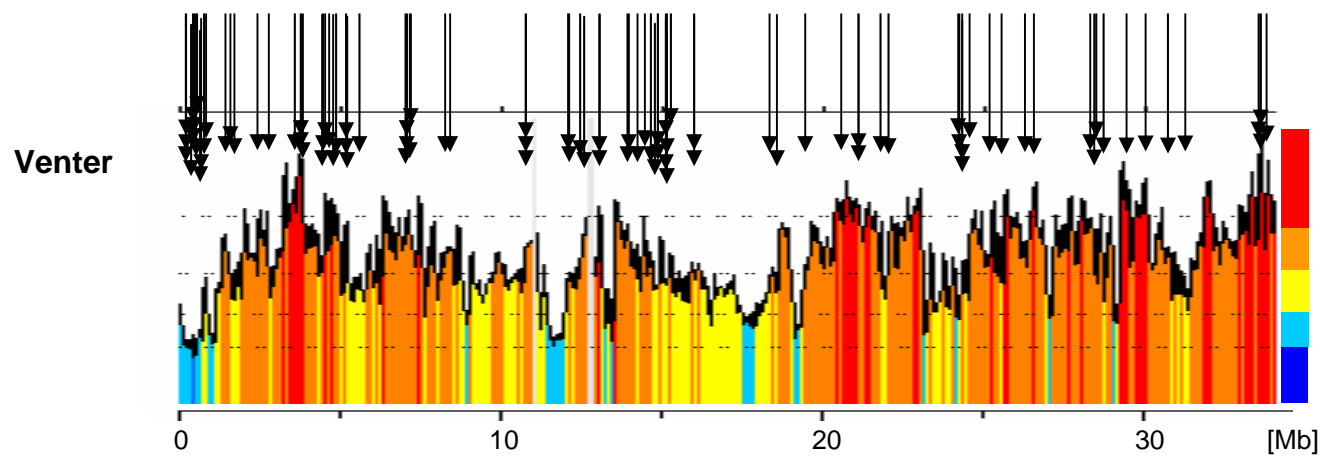

Supplement: Figure S3 — (0.07 MB PDF) [file pone.0005972.s003.pdf]

Suppl. Fig. S4

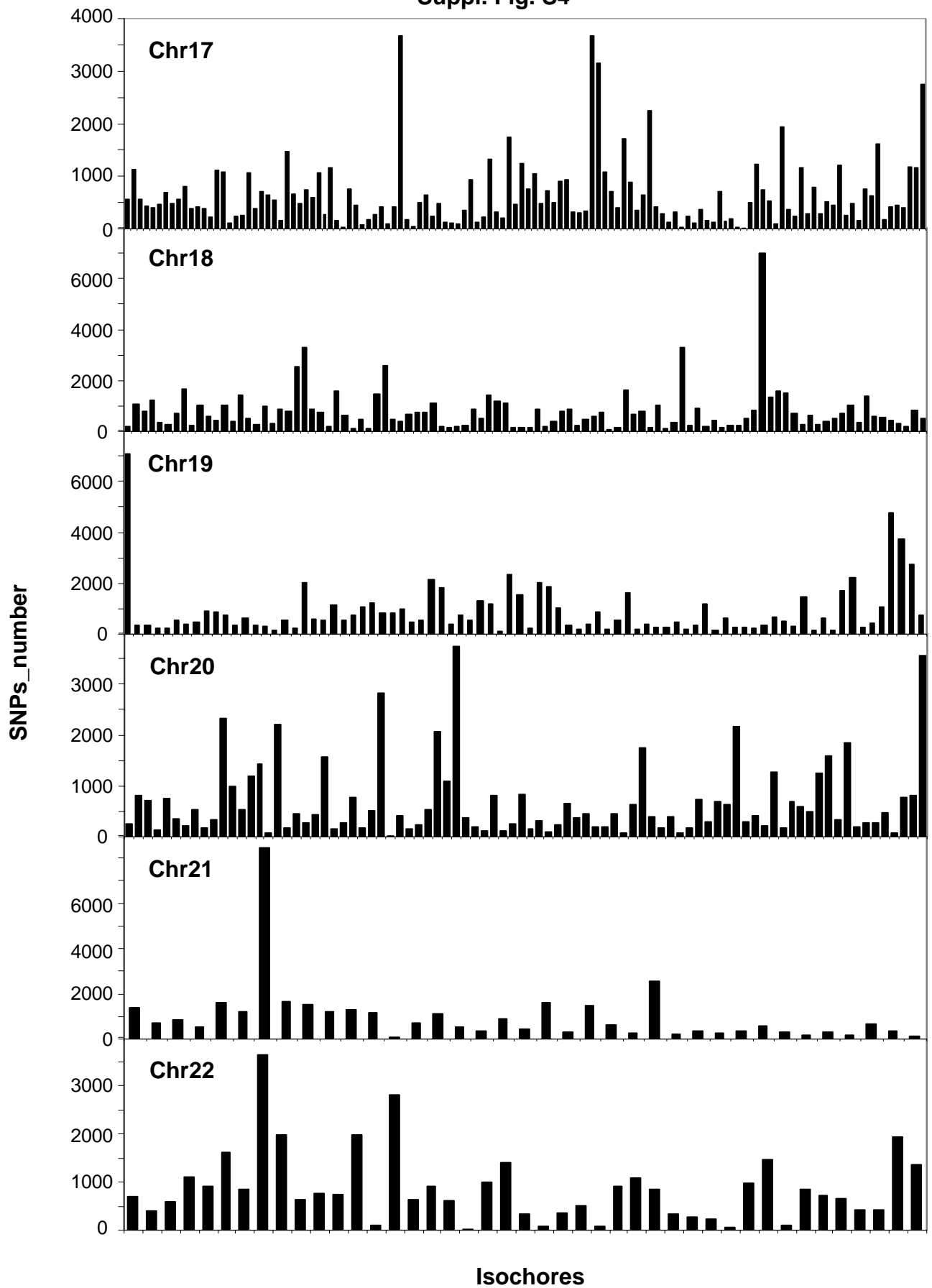

Supplement: Figure S4 — (0.04 MB PDF) [file pone.0005972.s004.pdf]

Suppl. Fig. S5A

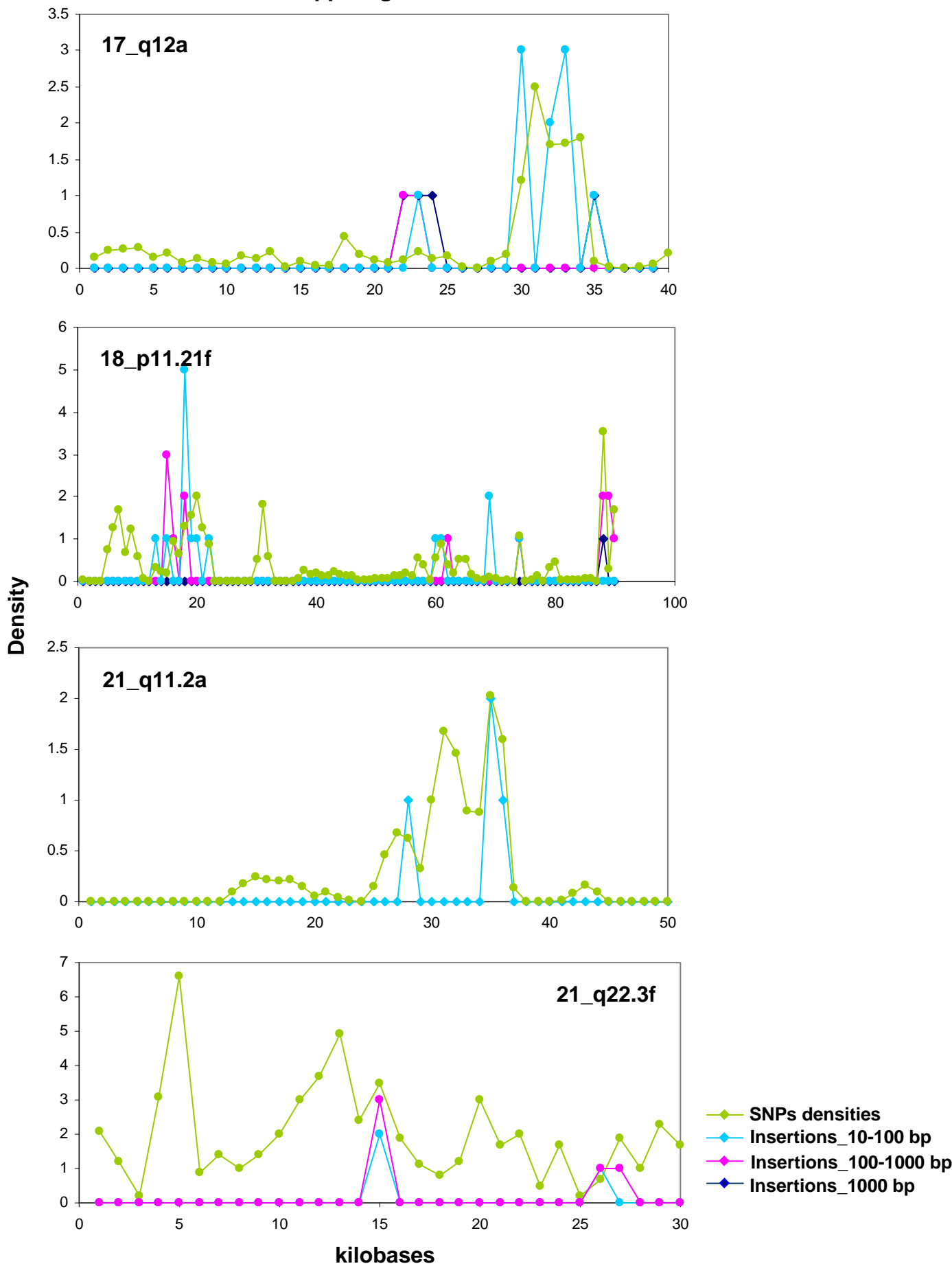

Suppl. Fig. S5B

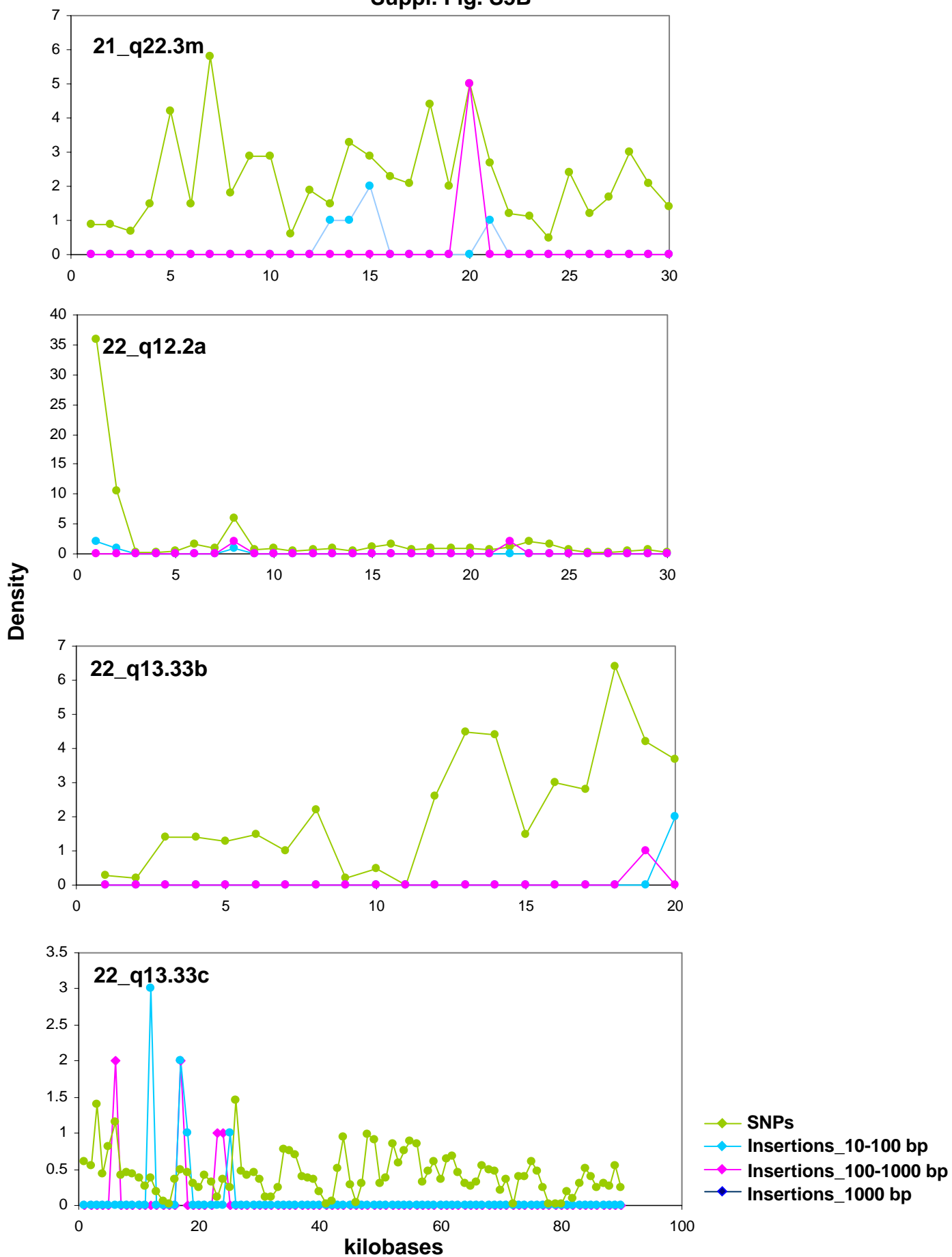

Supplement: Figure S5 — (0.08 MB PDF) [file pone.0005972.s005.pdf]
